# Loneliness Before and During the Covid-19 Pandemic: Associations with Chronic Illnesses and Relationship Quality

**DOI:** 10.1093/geroni/igab046.2742

**Published:** 2021-12-17

**Authors:** Jay Kayser, Jacqui Smith

**Affiliations:** 1 University of Michigan, Ann Arbor, Michigan, United States; 2 University of Michigan, University of Michigan, Michigan, United States

## Abstract

While self-reported loneliness generally declines after age 65, the likelihood of experiencing chronic illnesses increases. During the Covid-19 pandemic, social isolation measures have changed the social context of many people. We address three research questions: 1) What is the predictive strength of chronic illnesses, relationship quality, and their interaction on loneliness? 2) Has Covid-19 altered experienced loneliness relative to pre-pandemic? 3) Was loneliness during Covid-19 associated with the number of prior chronic illnesses in 2016? To answer these questions, we have analyzed data from participants in the Health and Retirement Study (HRS) included in the early 2020 release who also completed the 2016 wave (N = 1106). On average, in 2016, these participants were age 74.64 (SD = 6.66) and reported 2.57 (SD = 1.39) chronic illnesses. In 2016, unadjusted multiple regression models revealed that chronic illnesses (β = .38) and relationship quality (β = -.41) were associated with loneliness (R2 = .28). When covariates were added, these values were attenuated but remained statistically significant. In 2020 during the pandemic, 8% of these participants reported they often felt lonely and 26% reported feeling lonelier since the start of the Covid-19 pandemic. People who had more chronic illnesses in 2016 reported feeling lonelier in 2020 as did people whose relationships were poorer quality (p < .05). Further analyses with final data from HRS are needed to confirm these trends. These findings highlight the importance of having longitudinal information to identify individuals at high risk and most likely to benefit from interventions.

